# Dynamic evolution of postoperative hemodynamics in moyamoya angiopathy: a quantitative assessment of 4D Flow MRI and prognostic relevance

**DOI:** 10.3389/fneur.2025.1665883

**Published:** 2025-11-05

**Authors:** Chao Xia, Mingzhu Fu, Rui Tian, Yutao Ren, Xu Xu, Jinge Zhang, Chunchao Xia, Chao You, Na Hu, Su Lui, Rui Li, Yi Liu

**Affiliations:** ^1^Department of Neurosurgery, West China Hospital, Sichuan University, Chengdu, China; ^2^Department of Radiology, and Functional and Molecular Imaging Key Laboratory of Sichuan Province, West China Hospital, Sichuan University, Chengdu, China; ^3^Huaxi MR Research Center (HMRRC), West China Hospital, Sichuan University, Chengdu, China; ^4^Research Unit of Psychoradiology, Chinese Academy of Medical Sciences, Chengdu, China; ^5^Center for Biomedical Imaging Research, Department of Biomedical Engineering, Tsinghua University, Beijing, China; ^6^Department of Neurosurgery, The First Affiliated Hospital of Xi’an Jiaotong University, Xi’an, China; ^7^Department of Radiology, West China Hospital, Sichuan University, Chengdu, China; ^8^Sichuan Provincial Engineering Research Center of Intelligent Medical Imaging, West China Hospital, Sichuan University, Chengdu, China

**Keywords:** moyamoya angiopathy, 4D Flow MRI, hemodynamics, revascularization, prognostic relevance

## Abstract

**Purpose:**

The hemodynamic mechanisms underlying revascularization efficacy in moyamoya angiopathy (MMA) and their prognostic implications remain incompletely characterized. This study leverages four-dimensional flow magnetic resonance imaging (4D Flow MRI) to investigate longitudinal hemodynamic changes at the carotid siphons of MMA patients undergoing revascularization, and to evaluate their association with surgical outcomes.

**Methods:**

A prospective cohort of 35 consecutive MMA patients undergoing unilateral revascularization was enrolled at West China Hospital from July 2018 to January 2020. Using 4D Flow MRI, hemodynamic parameters, including mean/maximum flow, velocity, and wall shear stress, were quantified in the ipsilateral and contralateral carotid siphons at three timepoints: baseline, 1-week postoperative, and 1-year follow-up. Repeated-measures analysis of variance with Bonferroni correction was employed to compare longitudinal changes and correlate findings with 1-year clinical (excellent, good, and poor) and imaging (cerebral perfusion status and Matsushima collateralization grade) outcomes.

**Results:**

Baseline and 1-week postoperative assessments revealed that only velocity within contralateral carotid siphon significantly increased (mean velocity: from 20.52 [15.34–28.78] cm/s to 23.70 [16.01–39.06] cm/s, *p* = 0.026; maximum velocity: from 29.26 [19.68–38.39] cm/s to 33.22 [20.99–50.95] cm/s, *p* = 0.001). However, both carotid siphons demonstrated significant reductions in mean and maximum flow (ipsilateral: mean flow from 1.92 [0.65–3.53] mL/s to 1.31 [0.58–2.87] mL/s, *p* = 0.043, maximum flow from 2.61 [0.93–4.95] mL/s to 1.97 [0.89–3.90] mL/s, *p* = 0.036; contralateral: mean flow from 2.87 [0.91–4.12] mL/s to 2.14 [0.81–3.78] mL/s, *p* = 0.010) at 1-year follow-up. Lower contralateral siphon flow at follow-up correlated with “good” (but not “excellent”) clinical outcomes. Reduced flow in both siphons was associated with improved cerebral perfusion and robust collateralization (Matsushima grades A/B), whereas no changes were observed in patients with poor collaterals (Matsushima grade C).

**Conclusion:**

4D Flow MRI reveals delayed, bilateral hemodynamic remodeling in MMA at 1-year post-revascularization. These changes correlate with clinical improvement, enhanced perfusion, and collateral development, underscoring the technique utility in monitoring long-term cerebrovascular adaptation.

## 1 Introduction

Moyamoya angiopathy (MMA) is a rare, chronic cerebrovascular disease, characterized by progressive stenosis or occlusion of the internal carotid arteries (ICAs) and compensatory collateral vessel formation, which is typically staged using conventional angiography-based systems (e.g., the Suzuki scale) to classify its severity (1, 2). The etiology of MMA remains poorly understood, but abnormal hemodynamics are considered to play a crucial role in its pathogenesis (3, 4). Studies have shown that wall shear stress (WSS) in the distal region of ICAs is relatively low in MMA patients, suggesting that reduced WSS may contribute to the arterial pathology. Computational fluid dynamics studies of MMA have further demonstrated altered flow patterns in ICAs and adjacent communicating arteries (5), although these findings have been inconsistent across studies (6). Surgical revascularization, involving anastomosis of extracranial arteries to cortical vessels or indirect bypass techniques, aims to restore cerebral perfusion. The primary goal of revascularization is to improve cerebral hemodynamics, a crucial outcome that is often assessed by the presence or absence of perfusion improvement on postoperative imaging studies. However, the hemodynamic mechanisms underlying its therapeutic effects is unclear (7, 8).

Conventional imaging techniques for assessing hemodynamics (e.g., digital subtraction angiography [DSA] and computed tomography [CT]) typically involve radiation exposure, invasiveness, or indirect blood flow measurements (7). While DSA excellently depicts vascular anatomy and staging, and CT perfusion can quantify perfusion improvement, they lack the ability to comprehensively visualize complex flow patterns. Four-dimensional flow magnetic resonance imaging (4D Flow MRI) offers a non-invasive, direct technique for quantifying hemodynamic parameters throughout the cardiac cycle (9). This technique has been used to study various cerebrovascular and neurodegenerative conditions (10–14), demonstrating correlations between 4D Flow MRI-derived hemodynamic biomarkers and clinical features or outcomes. However, 4D Flow MRI has not been systematically explored in MMA.

Given the potential of 4D Flow MRI to provide detailed hemodynamic information, we aimed to apply this technique to MMA patients. Specifically, the carotid siphons, critical segments adjacent to the stenotic/occluded part of the ICAs in MMA, exhibit unique physiological flow characteristics essential to understanding disease pathophysiology. Their unique S-shaped geometry inherently generates complex helical flow patterns and flow separation, which predispose to pathologically low and oscillatory WSS (15, 16). These hemodynamic alterations create a vulnerable microenvironment that promotes endothelial dysfunction and atherosclerotic changes. In MMA, progressive morphological remodeling of the carotid siphons (e.g., luminal narrowing and tortuosity) further amplifies these disturbances, manifesting as elevated pressure drops and impaired cerebrovascular reserve (6, 7, 15, 17). Such hemodynamic parameters directly correlate with disease severity and clinical outcomes, providing mechanistic insights into why carotid siphon hemodynamics serve as critical biomarkers for assessing revascularization efficacy and long-term prognosis.

Therefore, we sought to investigate whether 4D Flow MRI could elucidate abnormal blood flow patterns through the carotid siphons. Furthermore, we aimed to determine whether the hemodynamic parameters measured by 4D Flow MRI are useful for assessing the outcomes of revascularization surgery.

## 2 Methods

### 2.1 Participants

This prospective observational study enrolled 35 patients with MMA from July 2018 to January 2020 at West China Hospital, Sichuan University. The study protocol was approved by the local institutional review board (No. 2018-219). Inclusion criteria were as follows: (1) diagnosis of moyamoya disease or moyamoya syndrome based on DSA according to established guidelines (18); (2) no prior revascularization; and (3) scheduled for unilateral revascularization surgery at the same hospital. Patients were required to undergo head imaging with 4D Flow MRI, CT perfusion, and DSA at three time points: before revascularization (baseline), 1 week after surgery, and 1 year after surgery (follow-up). Exclusion criteria included: (1) recent stroke (<3 months); (2) complete occlusion of bilateral carotid siphon based on DSA or 4D Flow MRI; (3) inadequate imaging quality; or (4) refusal of revascularization surgery. After enrollment, patients underwent unilateral combined revascularization surgery (details in Supplemental Materials). A power analysis based on our preliminary data from 20 patients indicated a minimum sample size of 26 to achieve 80% power (*α* = 0.05) using G*Power software (version 3.1.9.6, German).

### 2.2 Head imaging

All MRI examinations were performed by experienced technologists (>5 years of MRI experience) using a 3.0-T Skyra scanner (Siemens Medical Systems, Erlangen, Germany) with a 20-channel head coil. Scout imaging was initially performed to estimate blood flow velocity in the carotid siphons and the circle of Willis. The following parameters were used during Scout acquisition: echo time, 3.4 ms; repetition time, 21 ms; slice thickness, 6 mm; flip angle, 20°; field of view read, 208 mm; field of view phase, 100.0%; resolution matrix, 272 × 275; baseline velocity encoding, 80–100 cm/s; and acquisition time, 65 s. Based on Scout measurements, an optimally personalized “velocity encoding” value was defined for each patient for 4D Flow MRI of the carotid siphons. MRI was performed using a free-breathing, peripheral pulse-gated, multi-shot turbo field echo sequence, with the following parameters: echo time, 2.7 ms; repetition time, 45 ms; slice thickness, 1 mm; slice gap, 0 mm; flip angle, 10°; field of view read, 200 mm; field of view phase, 100.0%; resolution matrix, 256 × 216; and acquisition time, 19 min 50 s.

CT perfusion was performed using a 256-slice Revolution Apex scanner (GE Medical Systems, Brookfield, WI, USA) after injection of the contrast agent Iomeron® (Bracco, Milan, Italy) via a power injector at 4.5–5.0 mL/s. Imaging parameters were as follows: tube voltage, 80 kV; tube current, 300 mA; slice thickness, 5 mm; delay after injection of contrast agent, 5 s; cycle time, 2 s; number of slices, 640; and scan time, 50–55 s.

### 2.3 Analysis of 4D Flow MRI

Raw data from 4D Flow MRI were imported into VesselExplorer2 (V1.0.4.1, TSimaging Healthcare Co., Ltd., Beijing, China^1^). This software enables comprehensive analysis of 4D Flow MRI data, yielding key hemodynamic parameters and three-dimensionally dynamic visualization of blood flow patterns through streamline and pathline representations. Filtering thresholds were adjusted to maximize blood flow signal and minimize background noise. Velocity aliasing was minimized using anti-aliasing techniques. Following precise demarcation of carotid siphon segment boundaries, hemodynamic parameters (flow, velocity, and WSS) were systematically quantified within cross-sectional planes at the posterior siphon bend, with both mean and maximum values recorded for comprehensive hemodynamic profiling. WSS, which accounts for both circumferential and axial components, reflects the tangential force exerted on vascular endothelial cells by blood flow (19). All blood flow measurements were independently performed by an experienced neuroradiologist (CX) and an experienced neurosurgeon (YTR). Discrepancies were resolved through consultation with a senior neuroradiologist (SL).

### 2.4 Assessment of revascularization outcomes at follow-up

Clinical outcomes were evaluated through standardized preoperative and postoperative assessments conducted by experienced neurologists. At 1-year follow-up, clinical outcomes were evaluated using a three-tiered classification system: (1) “excellent” indicated complete resolution of preoperative neurological symptoms/signs (including dizziness, headache, limb weakness, memory impairment, and so on); (2) “good” referred to partial improvement with persistent but attenuated symptoms or signs; and (3) “poor” denoted unchanged or aggravated clinical manifestations compared to preoperative status. This evaluation combined patient self-reported symptom diaries with clinical examination findings, per established protocols referenced in prior literature (20). To ensure consistency, all assessments were performed blinded to imaging results, with discrepancies resolved through consensus discussions involving senior clinicians. This approach balances patient-centered outcomes with clinical objectivity while maintaining methodological rigor.

Imaging outcomes at 1-year follow-up were assessed independently by two experienced neuroradiologists (CX and MZF) under blinded conditions regarding clinical information. Discrepancies were resolved through consultation rounds with a senior neuroradiologist (SL). Cerebral perfusion was analyzed through serial CT perfusion scans (baseline vs. follow-up), quantified using the preinfarction staging system (21), with lower preinfarction stages reflecting improved perfusion. Collateral circulation was assessed by DSA and graded as A, B, or C using the Matsushima scale (Supplementary Figure S1) (22).

### 2.5 Statistical analysis

Data were statistically analyzed using SPSS 26.0 (IBM, Armonk, NY, USA), with results considered significant if associated with two-sided *p* < 0.05. Shapiro–Wilk test was performed before applying repeated-measures analysis of variance. Hemodynamic parameters in the ipsi- and contralateral carotid siphons were compared across three time points using repeated-measures analysis of variance for normally distributed data or Friedman test for non-normally distributed data, with Bonferroni corrections for multiple comparisons. Mauchly’s tests of sphericity were conducted to check assumptions. Parameters were also analyzed in subgroups defined by sex, age category (< or ≥18 years), or disease stage (categorized as “early” if in Suzuki stages I-II, “intermediate” if in Suzuki stages III-IV, or “advanced” if in Suzuki stages V-VI) (18, 23, 24). Associations of hemodynamic parameters with clinicodemographic characteristics or outcomes at follow-up were explored using repeated-measures analysis of variance with Bonferroni correction for multiple comparisons. The interclass correlation coefficient was used to evaluate interobserver consistency of blood flow measurements.

## 3 Results

**Figure 1:**
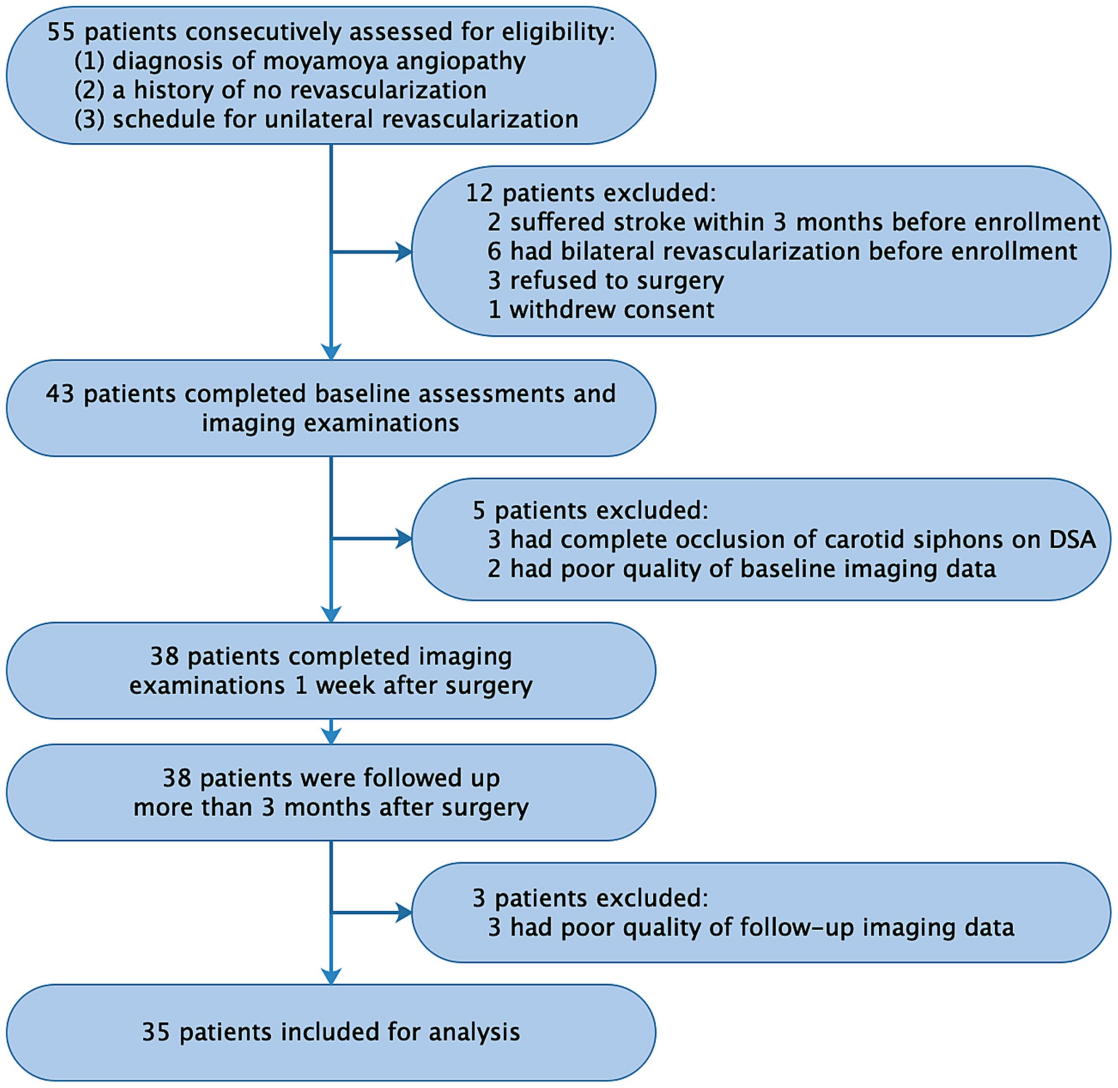
Flowchart of patient selection. DSA, digital subtraction angiography.

**Figure 2:**
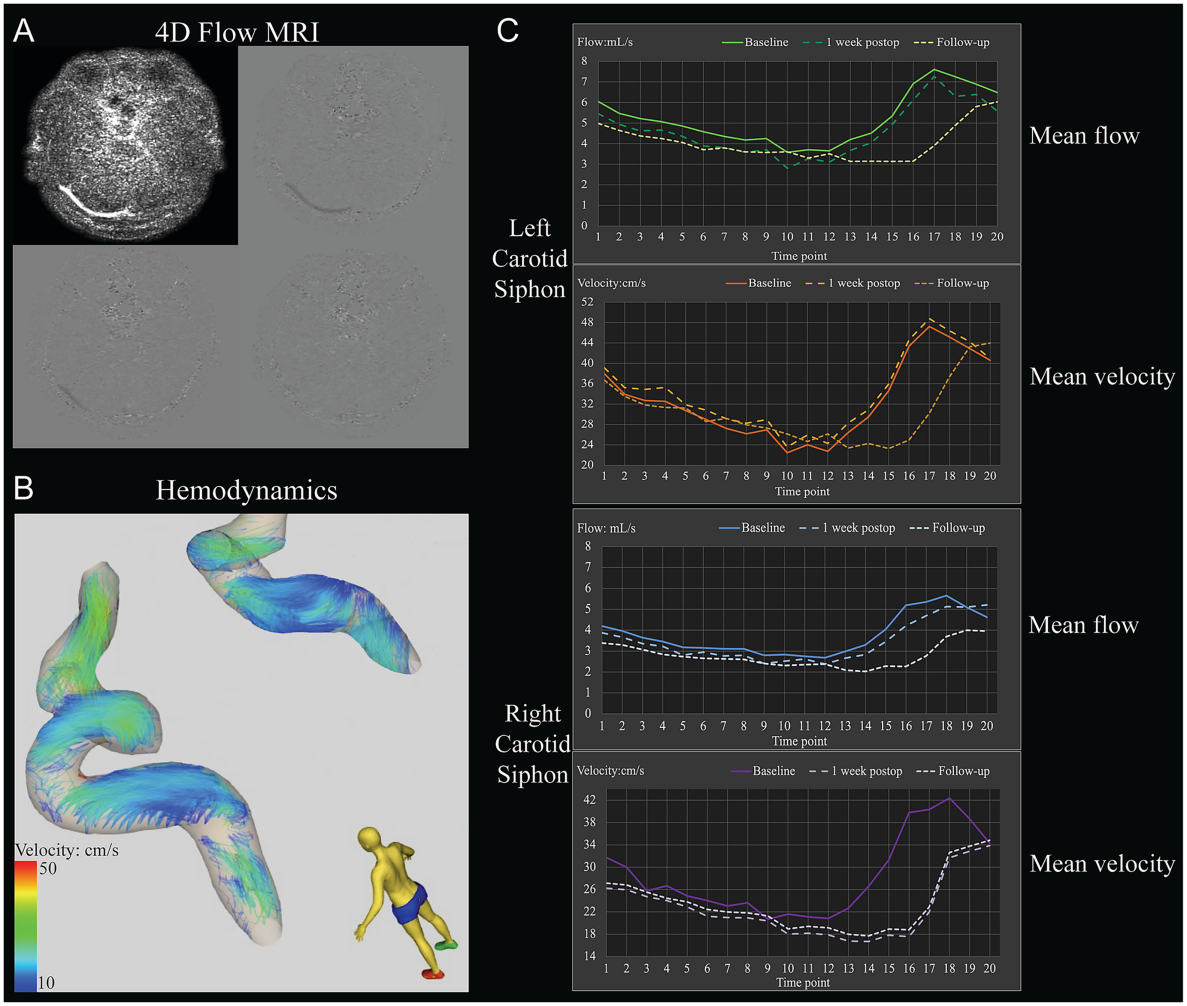
Example of the determination of hemodynamic parameters in the carotid siphons of a 31-year-old woman with moyamoya angiopathy who underwent left-side revascularization. **(A)** Raw 4D Flow MRI. **(B)** Pathline visualization of the bilateral carotid siphons. **(C)** Hemodynamic parameters included mean flow and mean velocity through the bilateral carotid siphons at different time points within one cardiac cycle before revascularization (baseline), 1 week after surgery, or 1 year after surgery (follow-up).

We analyzed 35 patients (15 males; mean age, 34.3 ± 17.7 years; range, 6–69 years), including 10 pediatric patients (<18 years) (Figure 1 and Table 1). All patients underwent unilateral revascularization and completed 1-year postoperative follow-up. At follow-up, 19 patients (54.3%) achieved “excellent” clinical outcomes (complete resolution of preoperative symptoms), while 16 (45.7%) were classified as “good” (persistent but attenuated symptoms) (Table 2). A representative case was shown in Figure 2.

**Table 1:** Clinicodemographic characteristics of the 35 participants at baseline.

Characteristics	Patients (*n* = 35)
Age, yr	34.3 ± 17.7
Female	20 (57.1)
Hypertension	9 (25.7)
Diabetes mellitus	1 (2.9)
Current smoker	5 (14.3)
Current drinker	2 (5.7)
Presentation of moyamoya angiopathy	
Ischemic	30 (85.7)
Hemorrhagic	5 (14.3)
Suzuki stage, surgical/contralateral side	
Early	5 (14.3)/12 (34.3)
Intermediate	19 (54.3)/16 (45.7)
Advanced	11 (31.4)/7 (20.0)
Preinfarction stage, surgical/contralateral side	
0	0 (0)/1 (2.9)
I	10 (28.6)/20 (57.1)
II	10 (28.6)/2 (5.7)
III	3 (8.6)/1 (2.9)
IV	12 (34.3)/11 (31.4)

Values are *n* (%) or mean ± SD.

**Table 2:** Outcomes of the 35 participants at 1-year follow-up.

Outcomes	Patients (*n* = 35)
Clinical condition	
Excellent	19 (54.3)
Good	16 (45.7)
Poor	0 (0)
Cerebral perfusion	
Better than at baseline	23 (65.7)
Not better than at baseline	12 (34.3)
Matsushima grade of collateralization	
A	12 (34.3)
B	13 (37.1)
C	10 (28.6)

Values are n (%).

### 3.1 Hemodynamic parameters via 4D Flow MRI

**Figure 3:**
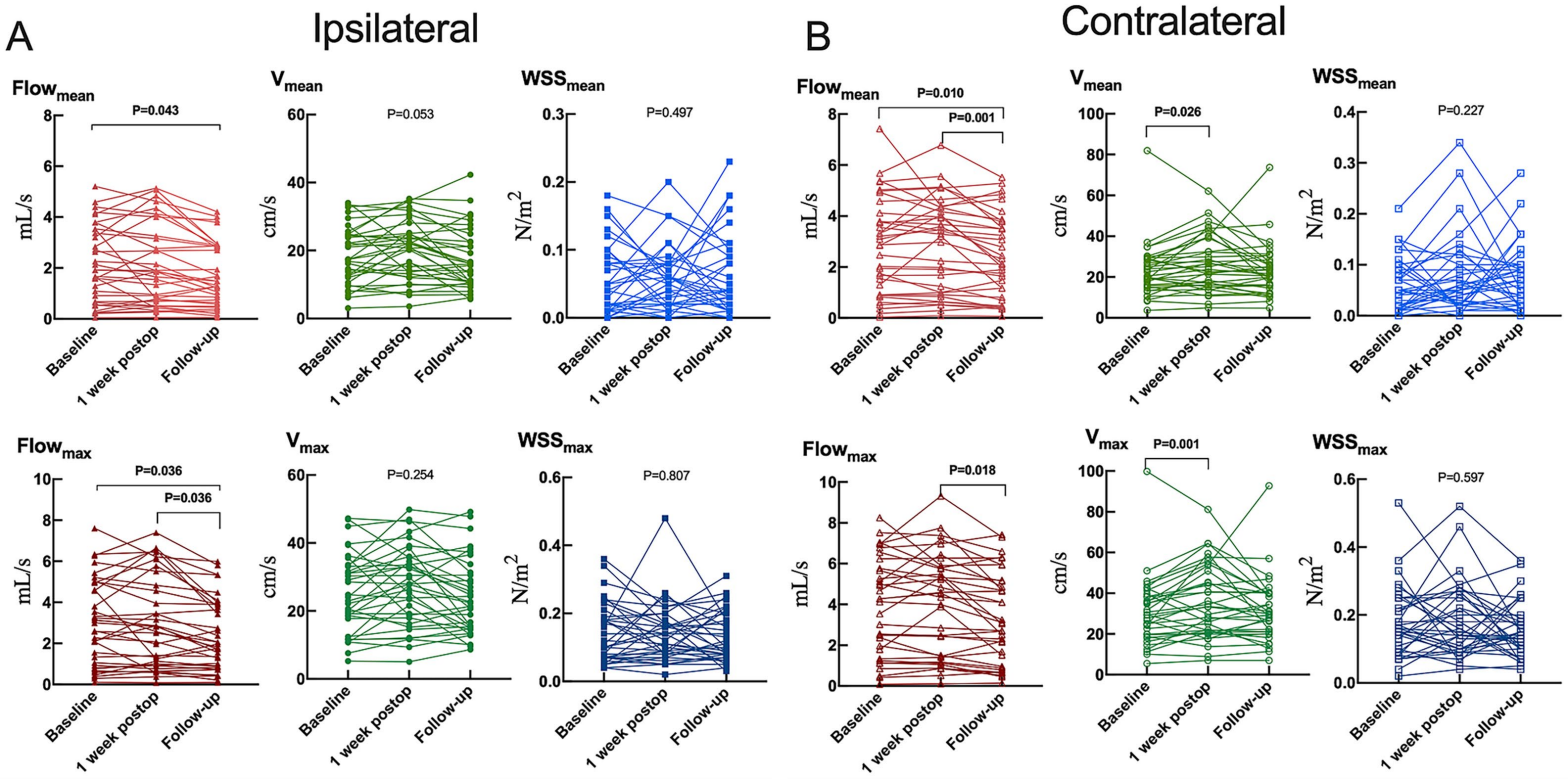
Hemodynamic parameters in the **(A)** ipsilateral or **(B)** contralateral carotid siphon before unilateral revascularization (baseline), at 1 week after surgery, or 1 year after surgery (follow-up). Flow_mean_, mean flow; Flow_max_, maximum flow; V_mean_, mean velocity; V_max_, maximum velocity; WSS_mean_, mean wall shear stress on vascular endothelium; WSS_max_, maximum wall shear stress on vascular endothelium. Results are plotted individually for the 35 patients in the study.

Baseline and 1-week postoperative assessments revealed that only velocity within contralateral carotid siphon significantly increased (mean: from 20.52 [15.34–28.78] cm/s to 23.70 [16.01–39.06] cm/s, *p* = 0.026; maximum: from 29.26 [19.68–38.39] cm/s to 33.22 [20.99–50.95] cm/s, *p* = 0.001) (Figure 3 and Supplementary Table S1). However, significant reductions were observed between baseline and 1 year postoperatively: (1) Ipsilateral siphon: Mean flow decreased from 1.92 (0.65–3.53) mL/s to 1.31 (0.58–2.87) mL/s (*p* = 0.043), and maximum flow decreased from 2.61 (0.93–4.95) mL/s to 1.97 (0.89–3.90) mL/s (*p* = 0.036); and (2) Contralateral siphon: Mean flow decreased from 2.87 (0.91–4.12) mL/s to 2.14 (0.81–3.78) mL/s (*p* = 0.010). Additionally, significant reductions were found between 1 week and 1 year postoperatively: (1) Ipsilateral siphon: Maximum flow decreased from 2.58 (0.93–4.83) mL/s to 1.97 (0.89–3.90) mL/s (*p* = 0.036); and (2) Contralateral siphon: Mean flow decreased from 3.29 (0.99–4.34) mL/s to 2.14 (0.81–3.78) mL/s (*p* = 0.001), and maximum flow decreased from 4.36 (1.40–5.80) mL/s to 3.06 (0.96–5.14) mL/s (*p* = 0.018). Interobserver reproducibility was excellent for flow measurements (interclass correlation coefficient > 0.83).

### 3.2 Subgroup analyses

Hemodynamic responses varied by sex, age, and disease stage. Females exhibited reductions in contralateral mean/maximum flow from 1 week to 1 year postoperatively (mean: from 3.25 [1.11–4.02] mL/s to 2.12 [0.65–3.20] mL/s, *p* = 0.008; maximum: from 4.18 [1.44–5.45] mL/s to 2.89 [0.97–4.51] mL/s, *p* = 0.013) and increases in contralateral maximum velocity from baseline to 1 week postoperatively (from 29.18 [18.28–29.23] cm/s to 31.55 [20.83–49.18] cm/s, *p* = 0.013). However, no significant changes were observed in males (Supplementary Table S2). Adults (>18 years) demonstrated significant reductions in bilateral mean flow (surgical side: from 1.89 [0.86–4.06] mL/s to 1.48 [0.90–2.92] mL/s, *p* = 0.049; contralateral side: from 3.59 [1.00–4.50] mL/s to 3.07 [1.32–3.85] mL/s, *p* = 0.006), ipsilateral maximum flow (from 2.82 [1.30–5.65] mL/s to 2.07 [1.33–4.04] mL/s, *p* = 0.033), and ipsilateral mean velocity (from 22.20 [15.07–30.21] cm/s to 16.19 [10.72–23.75] cm/s, *p* = 0.006), whereas pediatric patients showed no significant changes (Supplementary Table S3). For early-stage disease (Suzuki I-II), significant reductions were observed in mean/maximum ipsilateral flow (mean: from 4.40 [3.75–4.90] mL/s to 3.80 [2.89–4.12] mL/s, *p* = 0.034; maximum: from 5.95 [5.22–6.95] mL/s to 5.34 [3.67–5.90] mL/s, *p* = 0.034); for intermediate-stage disease (Suzuki III-IV), significant decreases in ipsilateral maximum flow (from 2.87 [2.08–4.83] mL/s to 2.38 [1.72–3.90] mL/s, *p* = 0.045), ipsilateral mean/maximum velocity (mean: from 22.20 [20.30–25.02] cm/s to 16.64 [11.20–22.61] cm/s, *p* = 0.006; maximum: from 30.45 [26.23–34.54] cm/s to 24.78 [15.55–30.54] cm/s, *p* = 0.028), and contralateral mean flow (from 3.10 [1.17–3.65] mL/s to 2.05 [1.25–3.20] mL/s, *p* = 0.014); and for advanced-stage disease (Suzuki V-VI), no hemodynamic changes detected (Supplementary Table S4–S6).

### 3.3 Surgical outcomes

**Figure 4:**
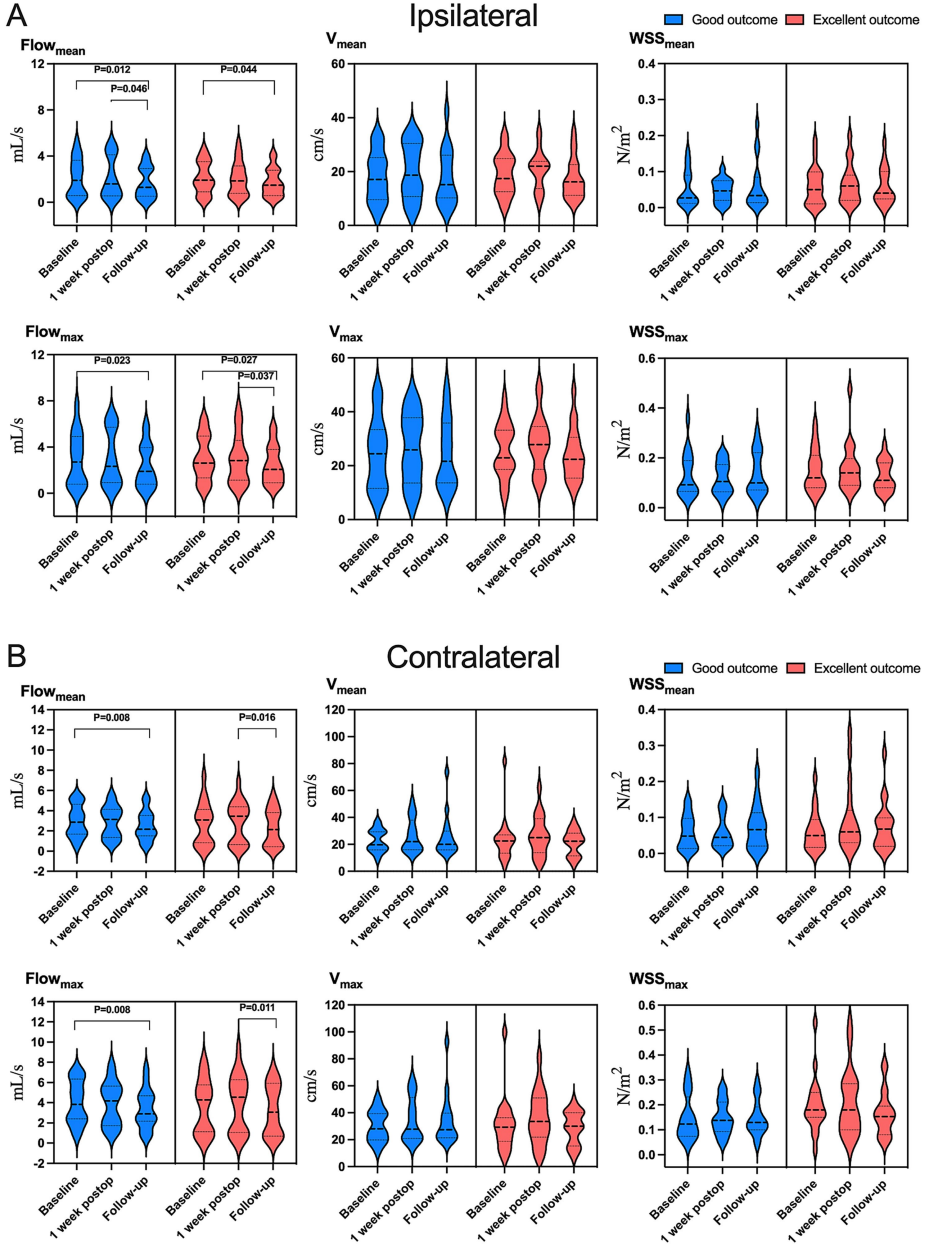
Variation in hemodynamic parameters in **(A)** ipsilateral or **(B)** contralateral carotid siphons in subsets of patients who were in “excellent” clinical condition (red) or “good” condition (blue) at follow-up (see Methods). Flow_mean_, mean flow; Flow_max_, maximum flow; V_mean_, mean velocity; V_max_, maximum velocity; WSS_mean_, mean wall shear stress on vascular endothelium; WSS_max_, maximum wall shear stress on vascular endothelium.

**Figure 5:**
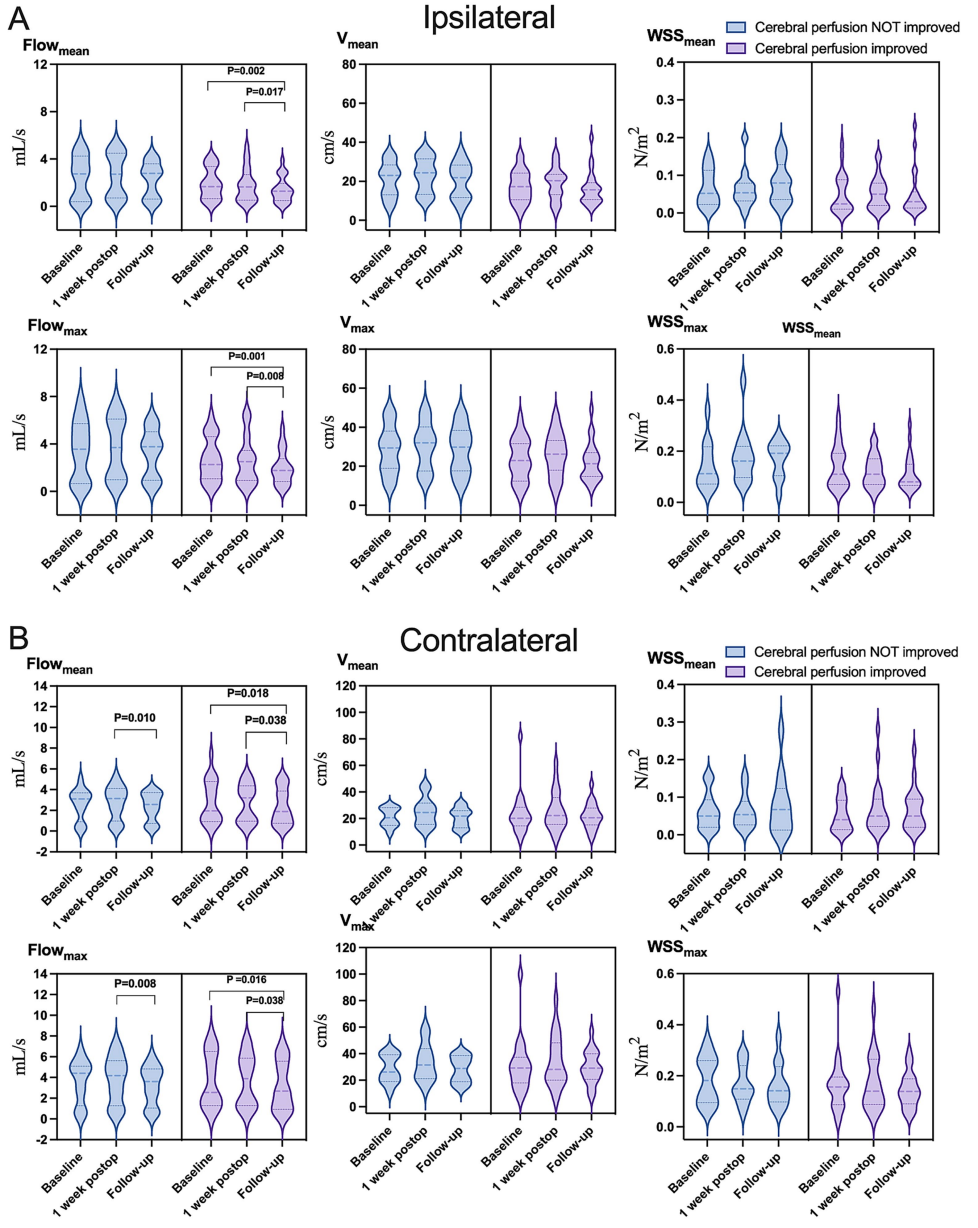
Variation in hemodynamic parameters in **(A)** ipsilateral or **(B)** contralateral carotid siphons in subsets of patients who showed improved cerebral perfusion (light purple) or not (light blue) at follow-up. Flow_mean_, mean flow; Flow_max_, maximum flow; V_mean_, mean velocity; V_max_, maximum velocity; WSS_mean_, mean wall shear stress on vascular endothelium; WSS_max_, maximum wall shear stress on vascular endothelium.

**Figure 6:**
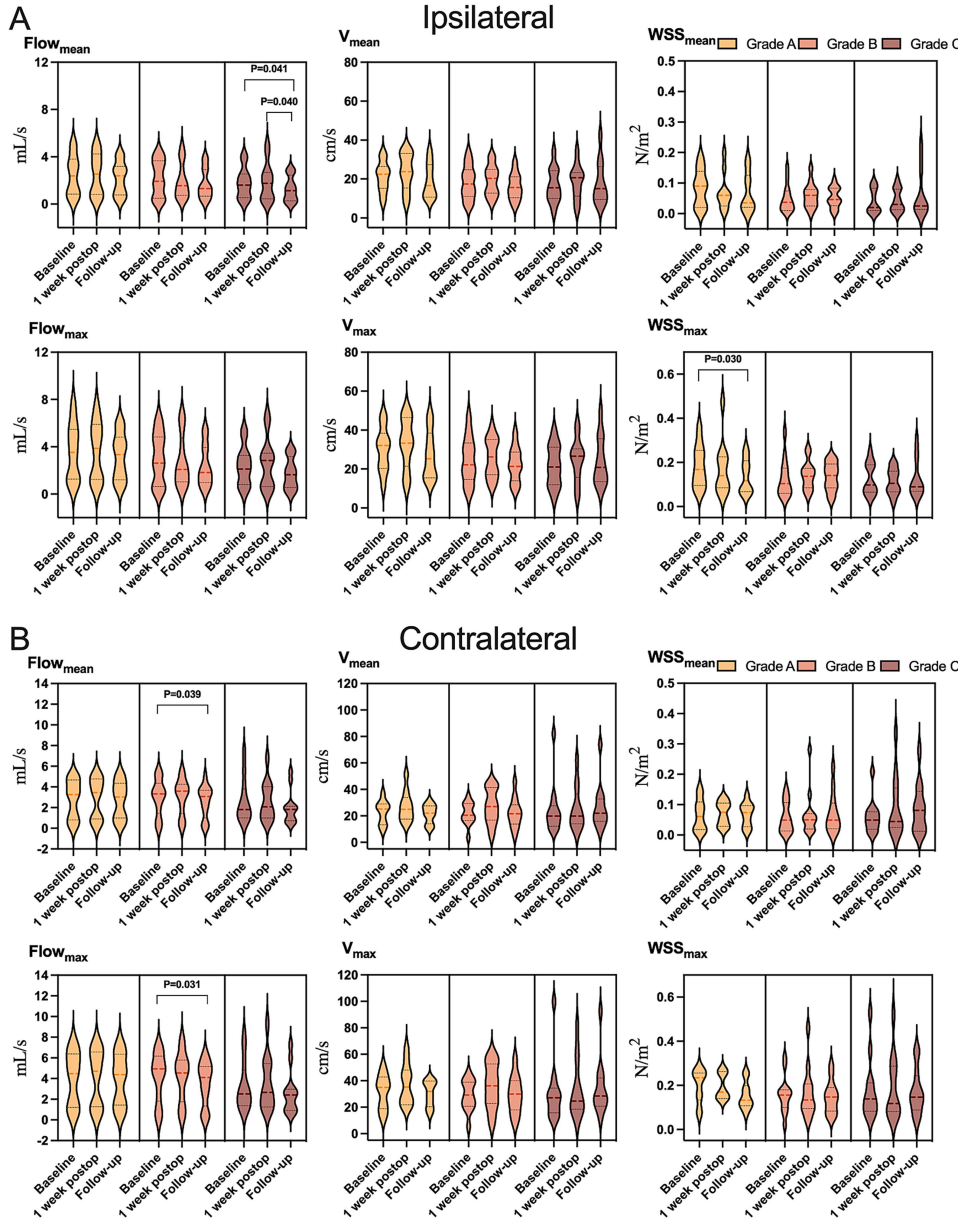
Variation in hemodynamic parameters in **(A)** ipsilateral or **(B)** contralateral carotid siphons in subsets of patients who showed postoperative collateralization of grade A (light yellow), grade B (yellow), or grade C (dark yellow) at follow-up. Flow_mean_, mean flow; Flow_max_, maximum flow; V_mean_, mean velocity; V_max_, maximum velocity; WSS_mean_, mean wall shear stress on vascular endothelium; WSS_max_, maximum wall shear stress on vascular endothelium.

Hemodynamic improvements correlated with clinical and imaging outcomes. Ipsilateral mean/maximum flow decreased significantly in both “excellent” and “good” outcome groups, while contralateral mean/maximum flow reductions were exclusive to the “good” outcome group (Figure 4 and Supplementary Table S7). Among 23 patients with improved preinfarction staging, ipsilateral flow/velocity decreased significantly; no changes were observed in the 12 patients without perfusion improvement (Figure 5 and Supplementary Table S8). Patients with robust collateralization (Matsushima grades A/B; *n* = 25) exhibited bilateral flow reductions, whereas those with poor collaterals (grade C; *n* = 10) showed no changes in flow or velocity (Figure 6 and Supplementary Table S9).

## 4 Discussion

Our study demonstrates that unilateral revascularization induces progressive, bilateral hemodynamic adaptation in MMA, with significant reductions in mean and maximum flow observed in both ipsi- and contralateral carotid siphons at 1-year follow-up. These changes were absent at 1 week postoperatively, suggesting delayed cerebrovascular remodeling rather than immediate flow redistribution. The association between reduced flow parameters and improved clinical outcomes, cerebral perfusion, and collateralization underscores the therapeutic relevance of these hemodynamic shifts. Notably, 4D Flow MRI enabled visualization of bilateral flow dynamics, offering mechanistic insights into revascularization efficacy and supporting its integration into postoperative surveillance protocols.

Our observation of diminished flow characteristics within the bilateral carotid siphons provides compelling experimental validation for computational fluid dynamic simulations that previously predicted analogous hemodynamic patterns in these vascular segments (25). The bilateral hemodynamic effects of unilateral revascularization likely reflect interconnected cerebrovascular adaptations. The circle of Willis and external carotid collateral networks, particularly the superficial temporal arteries, may mediate contralateral flow modulation, as hypothesized in computational models (25, 26). The persistent flow asymmetry (higher contralateral flow) at follow-up aligns with Suzuki stage-dependent stenosis severity where advanced ipsilateral occlusion necessitates complete perfusion shift to external carotid sources (15, 27). This compensatory mechanism may explain the lack of hemodynamic response in advanced-stage patients, as their cerebral circulation is already maximally redirected.

Subgroup analyses revealed critical age- and sex-related differences. Postmenopausal estrogen decline in females may reduce vascular adaptability, necessitating greater contralateral compensation (28). Conversely, children’s robust intrinsic collateral capacity likely delays postoperative hemodynamic adaptation (29), while adults derive clearer benefit from revascularization-induced external carotid augmentation. These findings suggest that surgical timing and laterality should be individualized based on age, sex, and disease stage. Our analysis reveals a significant correlation between reduced mean and maximum flow in both ipsi- and contralateral carotid siphons and concurrent improvements in clinical status, cerebral perfusion, and adaptive intracranial vascular remodeling. Prior investigations with limited cohort sizes have identified associations between revascularization outcomes and alternate hemodynamic indicators, such as pressure gradients or posterior communicating artery flow dynamics (6, 8, 30). However, these findings primarily focused on isolated parameters. Future multicenter studies with larger patient populations should employ comprehensive hemodynamic profiling to elucidate the complete spectrum of vascular response metrics that most robustly characterize cerebrovascular reactivity and surgical revascularization efficacy.

The stability of WSS parameters observed in our cohort aligns with findings from a cross-sectional analysis that similarly reported no significant associations between WSS and incident cerebrovascular events (31). This discrepancy with another studies demonstrating links between low WSS with ICA stenosis (5, 7) may reflect methodological heterogeneities in outcome measurement or inherent differences in hemodynamic stressors between vascular beds. Compensatory vascular remodeling, including reduced carotid siphon tortuosity (32, 33), may stabilize endothelial shear stress, thereby mitigating maladaptive responses like inflammation or platelet aggregation (8, 34–36). This hypothesis warrants investigation through longitudinal histological correlation studies.

Our findings must be interpreted with caution given several methodological limitations. First, the observational study design and relatively small sample size inherently limit statistical generalizability. This necessitates replication in larger prospective cohorts with longitudinal follow-up. Despite these limitations, the heterogeneity of our sample allowed us to demonstrate differential relationships between hemodynamic parameters and outcomes in different patient subgroups. Second, 4D Flow MRI’s limited spatial resolution (e.g., inability to assess vessels <1 mm) and potential motion artifacts may have influenced hemodynamic measurement. Nevertheless, our results substantiate the prognostic utility of this imaging modality in cerebrovascular disease characterization, underscoring the clinical imperative to advance technical development priorities. To fully realize its diagnostic potential in multicenter clinical trials, future research must emphasize the development of enhanced spatial resolution, accelerated acquisition protocols, and automated post-processing pipelines (37). While the VesselExplorer2 software lacks direct pressure computation capabilities, our approach aligns with fluid dynamics principles: future studies will integrate the acquired velocity fields and flow rates into the Navier–Stokes equations to computationally derive pressure distributions. Such integration would advance mechanistic understanding of how flow alterations translate into vascular stress and surgical outcome predictors, bridging a key gap in current MMA hemodynamic assessment paradigms.

## 5 Conclusion

This study establishes 4D Flow MRI as a powerful tool for monitoring long-term hemodynamic changes after MMA revascularization. The bilateral flow reductions observed at 1 year correlate with improved clinical and perfusion outcomes, supporting the technique’s use in evaluating surgical efficacy and guiding personalized management strategies. While WSS stability requires further investigation, our findings collectively advocate for integrating advanced hemodynamic imaging into MMA care pathways to optimize therapeutic decision-making.

## Data Availability

The raw data supporting the conclusions of this article will be made available by the authors, without undue reservation.

## Footnotes

^1^ http://www.tsimaging.net
