# Supplementary material for: Dynamic evolution of postoperative hemodynamics in moyamoya angiopathy: a quantitative assessment of 4D Flow MRI and prognostic relevance
Source: Front Neurol. 2025 Nov 5;16:1665883. doi: 10.3389/fneur.2025.1665883 (PMC12627057; doi:10.3389/fneur.2025.1665883)
Supplement: Supplementary file 1 [file Table_1.docx]

**Supplementary Material**

**Supplemental Methods**

*Surgical Procedures*

Following a comprehensive evaluation of clinical manifestations and radiological assessments, including digital subtraction angiography (DSA), computed tomography perfusion (CTP), and four-dimensional flow magnetic resonance imaging (4D Flow MRI), eligible patients were recommended for standard revascularization surgery. In this study, combined revascularization surgery was employed as the optimal surgical approach, specifically involving the anastomosis of the superficial temporal artery (STA) to the middle cerebral artery (MCA), coupled with encephaloduromyosynangiosis. All surgical procedures were meticulously performed by a senior neurosurgeon with extensive experience in standardized combined revascularization techniques.

Generally, the frontal and/or parietal branches of the STA were anastomosed to the cortical branches of the MCA using end-to-side anastomosis, facilitated by a single 10–0 absorbable nylon suture. The middle meningeal artery was carefully preserved throughout the procedure. Subsequently, the dura mater was incised radially and reflected with its outer layer positioned against the brain surface. The free end of the temporalis muscle was then positioned over the exposed cortex, securely sutured to the dura cuff, and affixed to the margin of the bone flap. Finally, the inferior aspect of the bone flap was appropriately contoured to accommodate both the STA and temporalis flap, ensuring optimal anatomical alignment and functional outcome.


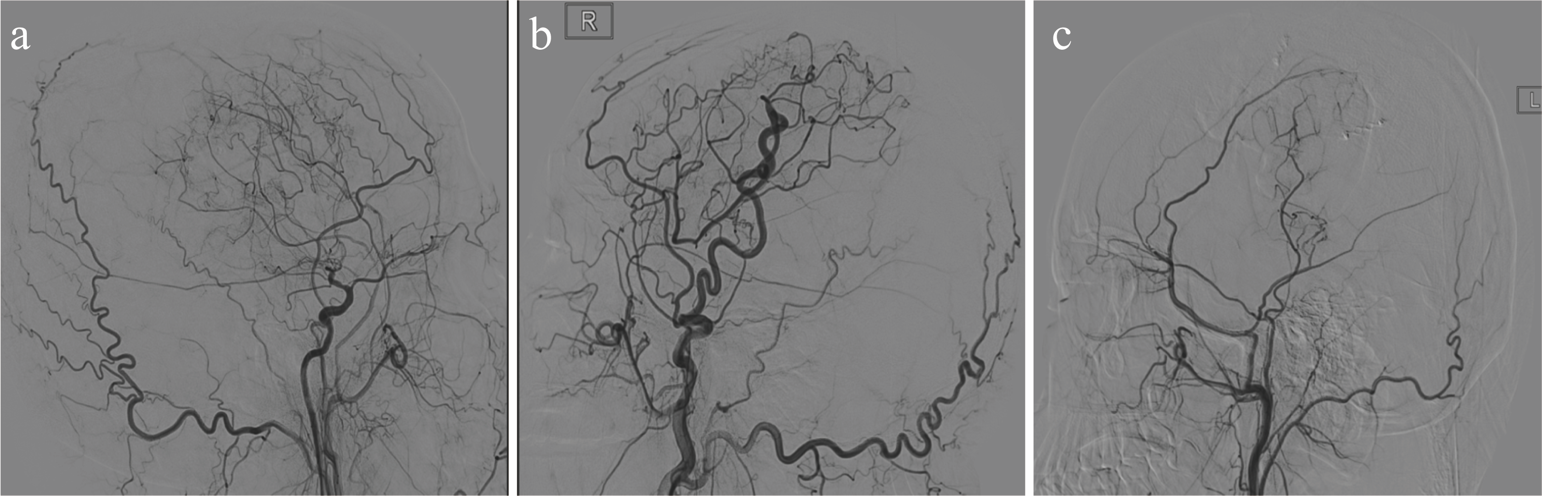


**FIGURE S1 Illustrations of collateral formation assessment post-revascularization using the Matsushima scale.**

a, Grade A: Extensive collateral formation with blood supply coverage exceeding two-thirds of the middle cerebral artery (MCA) territory.

b, Grade B: Moderate collateral formation, where the blood supply area spans between one-third and two-thirds of the MCA distribution.

c, Grade C: Limited collateral formation, characterized by blood supply coverage less than one-third of the MCA territory.

TABLE S1 Hemodynamics in both carotid siphons in patients before unilateral revascularization (baseline), at 1 week after surgery, or follow-up.

|  | Baseline | Postoperation | Follow-up | *P^a^* | *P^b^* | *P^c^* | *P^d^* |
| --- | --- | --- | --- | --- | --- | --- | --- |
| Surgical side |  |  |  |  |  |  |  |
| Flow_mean_ (mL/s) | 1.92 (0.65-3.53) | 1.85 (0.69-3.26) | 1.31 (0.58-2.87) | 0.020 | >0.999 | 0.043 | 0.050 |
| Flow_max_ (mL/s) | 2.61 (0.93-4.95) | 2.58 (0.93-4.83) | 1.97 (0.89-3.90) | 0.015 | >0.999 | 0.036 | 0.036 |
| V_mean_ (cm/s) | 17.41 (12.12-24.81) | 21.15 (13.20-25.23) | 16.04 (10.66-22.61) | 0.053 | - | - | - |
| V_max_ (cm/s) | 24.09 (17.77-33.18) | 27.81 (17.97-35.69) | 22.37 (15.09-31.31) | 0.254 | - | - | - |
| WSS_mean_ (N/m^2^) | 0.04 (0.01-0.09) | 0.05 (0.02-0.08) | 0.02 (0.04-0.09) | 0.497 | - | - | - |
| WSS_max_ (N/m^2^) | 0.11 (0.07-0.19) | 0.14 (0.08-0.18) | 0.11 (0.08-0.20) | 0.807 | - | - | - |
| Contralateral side |  |  |  |  |  |  |  |
| Flow_mean_ (mL/s) | 2.87 (0.91-4.12) | 3.29 (0.99-4.34) | 2.14 (0.81-3.78) | 0.001 | >0.999 | 0.010 | 0.001 |
| Flow_max_ (mL/s) | 4.13 (1.33-5.76) | 4.36 (1.40-5.80) | 3.06 (0.96-5.14) | 0.013 | >0.999 | 0.069 | 0.018 |
| V_mean_ (cm/s) | 20.52 (15.34-28.78) | 23.70 (16.01-39.06) | 21.63 (15.77-28.36) | 0.032 | 0.026 | 0.566 | 0.566 |
| V_max_ (cm/s) | 29.26 (19.68-38.39) | 33.22 (20.99-50.95) | 29.41 (20.95-39.98) | 0.002 | 0.001 | 0.219 | 0.219 |
| WSS_mean_ (N/m^2^) | 0.05 (0.02-0.09) | 0.05 (0.02-0.10) | 0.07 (0.02-0.10) | 0.227 | - | - | - |
| WSS_max_ (N/m^2^) | 0.16 (0.09-0.25) | 0.15 (0.10-0.26) | 0.14 (0.10-0.19) | 0.597 | - | - | - |

Values are median (IQR). Flow_mean_, mean flow; Flow_max_, maximum flow; V_mean_, mean velocity; V_max_, maximum velocity; WSS_mean_, mean wall shear stress on vascular endothelium; WSS_max_, maximum wall shear stress on vascular endothelium.

^a^ Three-time point comparisons; ^b^ Baseline vs. postoperative week 1; ^c^ Baseline vs. 1-year follow-up; ^d^ Postoperative week 1 vs. 1-year follow-up.

**TABLE S2 Hemodynamics in both carotid siphons at baseline, 1 week after surgery, and follow-up by sex group.**

|  | Male (N=15) | | | *P^a^* | *P^b^* | *P^c^* | *P^d^* | Female (N=20) | | | *P^a^* | *P^b^* | *P^c^* | *P^d^* |
| --- | --- | --- | --- | --- | --- | --- | --- | --- | --- | --- | --- | --- | --- | --- |
|  | Baseline | Postoperation | Follow-up |  |  |  |  | Baseline | Postoperation | Follow-up |  |  |  |  |
| Surgical side |  |  |  |  |  |  |  |  |  |  |  |  |  |  |
| Flow_mean_ (mL/s) | 0.91 (0.52-3.54) | 0.75 (0.43-2.77) | 0.64 (0.21-2.87) | 0.057 | - | - | - | 2.45 (1.59-3.49) | 2.02 (1.06-4.12) | 1.58 (1.23-2.89) | 0.259 | - | - | - |
| Flow_max_ (mL/s) | 1.33 (0.67-3.54) | 1.00 (0.65-3.95) | 0.89 (0.46-3.90) | 0.041 | >0.999 | 0.085 | 0.085 | 3.23 (2.14-4.87) | 2.85 (1.50-5.72) | 2.23 (1.73-3.88) | 0.259 | - | - | - |
| V_mean_ (cm/s) | 16.23 (8.17-22.16) | 16.68 (10.21-22.20) | 11.20 (9.29-16.70) | 0.165 | - | - | - | 19.80 (12.91-26.95) | 24.22 (13.81-30.42) | 17.73 (13.22-27.33) | 0.212 | - | - | - |
| V_max_ (cm/s) | 20.60 (11.64-31.12) | 23.81 (14.56-32.45) | 15.37 (12.96-25.77) | 0.819 | - | - | - | 26.63 (18.78-35.06) | 31.85 (18.81-38.99) | 24.84 (19.36-37.07) | 0.142 | - | - | - |
| WSS_mean_ (N/m^2^) | 0.020 (0.010-0.070) | 0.040 (0.010-0.080) | 0.030 (0.010-0.040) | 0.618 | - | - | - | 0.065 (0.013-0.115) | 0.060 (0.023-0.080) | 0.070 (0.025-0.133) | 0.627 | - | - | - |
| WSS_max_ (N/m^2^) | 0.100 (0.060-0.170) | 0.090 (0.060-0.170) | 0.080 (0.060-0.150) | 0.575 | - | - | - | 0.160 (0.080-0.225) | 0.150 (0.093-0.215) | 0.150 (0.083-0.220) | 0.831 | - | - | - |
| Contralateral side |  |  |  |  |  |  |  |  |  |  |  |  |  |  |
| Flow_mean_ (mL/s) | 3.32 (0.91-4.93) | 3.67 (0.99-4.39) | 3.49 (0.81-4.19) | 0.436 | - | - | - | 2.86 (0.99-3.71) | 3.25 (1.11-4.02) | 2.12 (0.65-3.20) | 0.001 | >0.999 | 0.008 | 0.001 |
| Flow_max_ (mL/s) | 4.67 (1.33-6.56) | 5.24 (1.40-6.27) | 4.69 (0.96-5.95) | 0.627 | - | - | - | 3.84 (1.38-5.23) | 4.18 (1.44-5.45) | 2.89 (0.97-4.51) | 0.010 | >0.999 | 0.053 | 0.013 |
| V_mean_ (cm/s) | 20.13 (16.33-29.41) | 23.70 (15.08-39.06) | 20.41 (15.83-30.70) | 0.165 | - | - | - | 23.05 (14.88-28.71) | 23.77 (16.05-37.92) | 23.66 (13.15-28.09) | 0.086 | - | - | - |
| V_max_ (cm/s) | 29.26 (19.93-38.23) | 33.22 (21.81-55.84) | 27.93 (22.05-39.96) | 0.074 | - | - | - | 29.18 (18.28-29.23) | 31.55 (20.83-49.18) | 31.03 (20.00-40.30) | 0.015 | 0.013 | 0.173 | >0.999 |
| WSS_mean_ (N/m^2^) | 0.050 (0.010-0.100) | 0.050 (0.020-0.100) | 0.040 (0.020-0.100) | 0.796 | - | - | - | 0.060 (0.020-0.088) | 0.055 (0.023-0.113) | 0.070 (0.040-0.128) | 0.149 | - | - | - |
| WSS_max_ (N/m^2^) | 0.160 (0.090-0.250) | 0.150 (0.100-0.260) | 0.130 (0.100-0.180) | 0.135 | - | - | - | 0.155 (0.088-0.210) | 0.150 (0.093-0.258) | 0.150 (0.100-0.238) | 0.525 | - | - | - |

Values are median (IQR). Flow_mean_, mean flow; Flow_max_, maximum flow; V_mean_, mean velocity; V_max_, maximum velocity; WSS_mean_, mean wall shear stress on vascular endothelium; WSS_max_, maximum wall shear stress on vascular endothelium.

^a^ Three-time point comparisons; ^b^ Baseline vs. postoperative week 1; ^c^ Baseline vs. 1-year follow-up; ^d^ Postoperative week 1 vs. 1-year follow-up.

**TABLE S3 Hemodynamics in both carotid siphons at baseline, 1 week after surgery, and follow-up by age category.**

|  | Adult (N=25) | | | *P^a^* | *P^b^* | *P^c^* | *P^d^* | Child (N=10) | | | *P^a^* | *P^b^* | *P^c^* | *P^d^* |
| --- | --- | --- | --- | --- | --- | --- | --- | --- | --- | --- | --- | --- | --- | --- |
|  | Baseline | Postoperation | Follow-up |  |  |  |  | Baseline | Postoperation | Follow-up |  |  |  |  |
| Surgical side |  |  |  |  |  |  |  |  |  |  |  |  |  |  |
| Flow_mean_ (mL/s) | 2.26 (1.00-3.67) | 1.89 (0.86-4.06) | 1.48 (0.90-2.92) | 0.003 | >0.999 | 0.003 | 0.049 | 1.13 (0.45-2.26) | 0.72 (0.32-2.68) | 0.69 (0.29-1.97) | 0.407 | - | - | - |
| Flow_max_ (mL/s) | 3.23 (1.44-5.12) | 2.82 (1.30-5.65) | 2.07 (1.33-4.04) | 0.002 | >0.999 | 0.002 | 0.033 | 1.45 (0.65-3.18) | 0.97 (0.58-3.45) | 0.96 (0.45-2.70) | 0.407 | - | - | - |
| V_mean_ (cm/s) | 21.08 (12.56-25.21) | 22.20 (15.07-30.21) | 16.19 (10.72-23.75) | 0.008 | 0.359 | 0.359 | 0.006 | 15.25 (9.74-24.23) | 14.72 (9.40-22.43) | 12.71 (9.50-23.87) | 0.670 | - | - | - |
| V_max_ (cm/s) | 28.51 (18.58-34.56) | 32.45 (20.14-37.29) | 22.69 (15.36-32.53) | 0.080 | - | - | - | 19.90 (11.39-30.64) | 21.49 (12.02-28.44) | 17.80 (12.93-32.22) | 0.741 | - | - | - |
| WSS_mean_ (N/m^2^) | 0.040 (0.010-0.095) | 0.060 (0.035-0.080) | 0.040 (0.015-0.095) | 0.523 | - | - | - | 0.035 (0.018-0.085) | 0.020 (0.018-0.065) | 0.025 (0.018-0.095) | 0.918 | - | - | - |
| WSS_max_ (N/m^2^) | 0.140 (0.070-0.210) | 0.160 (0.090-0.210) | 0.140 (0.075-0.205) | 0.927 | - | - | - | 0.090 (0.058-0.188) | 0.080 (0.058-0.130) | 0.100 (0.078-0.148) | 0.282 | - | - | - |
| Contralateral side |  |  |  |  |  |  |  |  |  |  |  |  |  |  |
| Flow_mean_ (mL/s) | 3.32 (1.29-4.76) | 3.59 (1.00-4.50) | 3.07 (1.32-3.85) | 0.007 | 0.774 | 0.143 | 0.006 | 1.74 (0.73-2.88) | 1.95 (0.76-3.40) | 1.41 (0.64-2.11) | 0.050 | - | - | - |
| Flow_max_ (mL/s) | 4.67 (1.85-6.40) | 4.84 (1.43-6.36) | 4.10 (1.94-5.56) | 0.069 | - | - | - | 2.23 (0.94-3.58) | 2.45 (1.01-4.29) | 1.83 (0.84-2.80) | 0.150 | - | - | - |
| V_mean_ (cm/s) | 24.81 (18.12-29.28) | 27.46 (17.87-39.87) | 22.38 (15.23-29.53) | 0.102 | - | - | - | 16.08 (12.77-22.92) | 16.71 (13.77-25.36) | 19.80 (14.36-26.14) | 0.273 | - | - | - |
| V_max_ (cm/s) | 34.79 (23.45-40.45) | 40.52 (23.46-54.04) | 30.79 (21.58-40.06) | 0.009 | 0.009 | >0.999 | 0.102 | 19.85 (16.67-28.43) | 21.32 (19.35-33.71) | 27.31 (18.75-33.50) | 0.067 | - | - | - |
| WSS_mean_ (N/m^2^) | 0.070 (0.020-0.105) | 0.060 (0.025-0.110) | 0.070 (0.025-0.100) | 0.531 | - | - | - | 0.030 (0.008-0.055) | 0.040 (0.018-0.100) | 0.065 (0.010-0.115) | 0.202 | - | - | - |
| WSS_max_ (N/m^2^) | 0.160 (0.120-0.250) | 0.160 (0.105-0.255) | 0.150 (0.115-0.190) | 0.397 | - | - | - | 0.125 (0.063-0.220) | 0.120 (0.073-0.283) | 0.110 (0.058-0.208) | 0.717 | - | - | - |

Values are median (IQR). Flow_mean_, mean flow; Flow_max_, maximum flow; V_mean_, mean velocity; V_max_, maximum velocity; WSS_mean_, mean wall shear stress on vascular endothelium; WSS_max_, maximum wall shear stress on vascular endothelium.

^a^ Three-time point comparisons; ^b^ Baseline vs. postoperative week 1; ^c^ Baseline vs. 1-year follow-up; ^d^ Postoperative week 1 vs. 1-year follow-up.

**TABLE S4 Hemodynamics in both carotid siphons at baseline, 1 week after surgery, and follow-up in patients in the early stage.**

|  | Early stage^*^ | | | *P^a^* | *P^b^* | *P^c^* | *P^d^* |
| --- | --- | --- | --- | --- | --- | --- | --- |
|  | Baseline | Postoperation | Follow-up |  |  |  |  |
| Surgical side |  |  |  |  |  |  |  |
| Flow_mean_ (mL/s) | 4.40 (3.75-4.90) | 4.62 (3.64-5.11) | 3.80 (2.89-4.12) | 0.022 | >0.999 | 0.034 | 0.081 |
| Flow_max_ (mL/s) | 5.95 (5.22-6.95) | 6.21 (5.05-6.98) | 5.34 (3.67-5.90) | 0.022 | >0.999 | 0.034 | 0.081 |
| V_mean_ (cm/s) | 29.55 (25.21-32.10) | 34.37 (31.71-34.90) | 30.26 (23.75-38.53) | 0.091 | - | - | - |
| V_max_ (cm/s) | 39.69 (33.36-46.08) | 46.29 (42.48-48.35) | 44.21 (32.53-48.46) | 0.247 | - | - | - |
| WSS_mean_ (N/m^2^) | 0.120 (0.090-0.170) | 0.080 (0.065-0.175) | 0.110 (0.075-0.205) | 0.846 | - | - | - |
| WSS_max_ (N/m^2^) | 0.230 (0.175-0.325) | 0.220 (0.180-0.360) | 0.220 (0.190-0.285) | 0.819 | - | - | - |
| Contralateral side |  |  |  |  |  |  |  |
| Flow_mean_ (mL/s) | 4.96 (3.85-5.37) | 4.37 (4.01-5.13) | 4.03 (2.98-4.96) | 0.205 | - | - | - |
| Flow_max_ (mL/s) | 6.67 (5.37-7.03) | 6.09 (5.55-7.28) | 5.55 (4.25-6.53) | 0.368 | - | - | - |
| V_mean_ (cm/s) | 29.27 (20.20-34.81) | 35.75 (23.15-46.53) | 29.09 (21.33-34.84) | 0.125 | - | - | - |
| V_max_ (cm/s) | 40.39 (26.46-45.52) | 50.38 (30.85-63.13) | 39.80 (28.65-45.89) | 0.076 | - | - | - |
| WSS_mean_ (N/m^2^) | 0.055 (0.013-0.125) | 0.065 (0.020-0.120) | 0.100 (0.060-0.153) | 0.290 | - | - | - |
| WSS_max_ (N/m^2^) | 0.195 (0.150-0.258) | 0.210 (0.140-0.270) | 0.195 (0.125-0.258) | 0.649 | - | - | - |

Values are median (IQR). Flow_mean_, mean flow; Flow_max_, maximum flow; V_mean_, mean velocity; V_max_, maximum velocity; WSS_mean_, mean wall shear stress on vascular endothelium; WSS_max_, maximum wall shear stress on vascular endothelium.

^*^Depending on angiographic characteristics of baseline digital subtraction angiography, the early stage was defined as Suzuki stages Ⅰ and Ⅱ.

^a^ Three-time point comparisons; ^b^ Baseline vs. postoperative week 1; ^c^ Baseline vs. 1-year follow-up; ^d^ Postoperative week 1 vs. 1-year follow-up.

**TABLE S5 Hemodynamics in both carotid siphons at baseline, 1 week after surgery, and follow-up in patients in the intermediate stage.**

|  | Intermediate stage^*^ | | | *P^a^* | *P^b^* | *P^c^* | *P^d^* |
| --- | --- | --- | --- | --- | --- | --- | --- |
|  | Baseline | Postoperation | Follow-up |  |  |  |  |
| Surgical side |  |  |  |  |  |  |  |
| Flow_mean_ (mL/s) | 2.13 (1.60-3.21) | 1.91 (1.55-3.26) | 1.66 (1.21-2.87) | 0.065 | - | - | - |
| Flow_max_ (mL/s) | 3.14 (2.13-4.57) | 2.87 (2.08-4.83) | 2.38 (1.72-3.90) | 0.019 | >0.999 | 0.045 | 0.045 |
| V_mean_ (cm/s) | 21.08 (13.79-24.19_ | 22.20 (20.30-25.02) | 16.64 (11.20-22.61) | 0.006 | >0.999 | 0.069 | 0.006 |
| V_max_ (cm/s) | 28.51 (19.59-31.55) | 30.45 (26.23-34.54) | 24.78 (15.55-30.54) | 0.029 | 0.223 | >0.999 | 0.028 |
| WSS_mean_ (N/m^2^) | 0.050 (0.010-0.090) | 0.060 (0.020-0.080) | 0.040 (0.020-0.090) | 0.128 | - | - | - |
| WSS_max_ (N/m^2^) | 0.140 (0.100-0.180) | 0.140 (0.090-0.170) | 0.130 (0.080-0.180) | 0.899 | - | - | - |
| Contralateral side |  |  |  |  |  |  |  |
| Flow_mean_ (mL/s) | 2.66 (1.39-3.29) | 3.10 (1.17-3.65) | 2.05 (1.25-3.20) | 0.015 | >0.999 | 0.155 | 0.014 |
| Flow_max_ (mL/s) | 3.28 (1.94-4.78) | 3.94 (1.70-5.08) | 2.70 (1.81-4.51) | 0.087 | - | - | - |
| V_mean_ (cm/s) | 21.53 (15.46-27.02) | 25.08 (16.59-29.51) | 22.01 (15.79-25.87) | 0.368 | - | - | - |
| V_max_ (cm/s) | 29.36 (19.01-36.09) | 33.34 (21.83-44.29) | 29.67 (21.40-38.57) | 0.105 | - | - | - |
| WSS_mean_ (N/m^2^) | 0.060 (0.020-0.090) | 0.060 (0.033-0.130) | 0.060 (0.020-0.090) | 0.337 | - | - | - |
| WSS_max_ (N/m^2^) | 0.155 (0.115-0.210) | 0.155 (0.100-0.275) | 0.145 (0.113-0.178) | 0.802 | - | - | - |

Values are median (IQR). Flow_mean_, mean flow; Flow_max_, maximum flow; V_mean_, mean velocity; V_max_, maximum velocity; WSS_mean_, mean wall shear stress on vascular endothelium; WSS_max_, maximum wall shear stress on vascular endothelium.

^*^Depending on angiographic characteristics of baseline digital subtraction angiography, the intermediate stages was defined as Suzuki stages Ⅲ and Ⅳ.

^a^ Three-time point comparisons; ^b^ Baseline vs. postoperative week 1; ^c^ Baseline vs. 1-year follow-up; ^d^ Postoperative week 1 vs. 1-year follow-up.

**TABLE S6 Hemodynamics in both carotid siphons at baseline, 1 week after surgery, and follow-up in patients in the advanced stage.**

|  | Advanced stage^*^ | | | *P^a^* | *P^b^* | *P^c^* | *P^d^* |
| --- | --- | --- | --- | --- | --- | --- | --- |
|  | Baseline | Postoperation | Follow-up |  |  |  |  |
| Surgical side |  |  |  |  |  |  |  |
| Flow_mean_ (mL/s) | 0.52 (0.25-0.65) | 0.50 (0.30-0.75) | 0.46 (0.20-0.64) | 0.614 | - | - | - |
| Flow_max_ (mL/s) | 0.67 (0.39-0.93) | 0.77 (0.58-1.00) | 0.74 (0.41-0.91) | 0.913 | - | - | - |
| V_mean_ (cm/s) | 9.51 (6.85-16.23) | 10.21 (7.57-13.69) | 10.07 (7.04-13.87) | 0.529 | - | - | - |
| V_max_ (cm/s) | 11.64 (10.76-20.60) | 14.56 (11.68-18.69) | 14.81 (10.07-20.63) | 0.148 | - | - | - |
| WSS_mean_ (N/m^2^) | 0.020 (0.010-0.030) | 0.020 (0.000-0.060) | 0.020 (0.010-0.040) | 0.975 | - | - | - |
| WSS_max_ (N/m^2^) | 0.060 (0.050-0.070) | 0.070 (0.050-0.110) | 0.080 (0.050-0.100) | 0.461 | - | - | - |
| Contralateral side |  |  |  |  |  |  |  |
| Flow_mean_ (mL/s) | 0.83 (0.18-0.91) | 0.64 (0.29-0.91) | 0.44 (0.37-0.81) | 0.163 | - | - | - |
| Flow_max_ (mL/s) | 1.11 (0.43-1.24) | 1.05 (0.51-1.17) | 0.68 (0.45-0.89) | 0.368 | - | - | - |
| V_mean_ (cm/s) | 13.44 (8.79-19.61) | 13.85 (11.25-16.16) | 11.95 (10.13-16.18) | 0.565 | - | - | - |
| V_max_ (cm/s) | 18.11 (11.20-22.64) | 20.78 (17.47-21.86) | 18.17 (12.83-20.95) | 0.156 | - | - | - |
| WSS_mean_ (N/m^2^) | 0.030 (0.020-0.050) | 0.030 (0.020-0.060) | 0.020 (0.010-0.060) | 0.857 | - | - | - |
| WSS_max_ (N/m^2^) | 0.080 (0.070-0.180) | 0.090 (0.050-0.160) | 0.070 (0.050-0.130) | 0.964 | - | - | - |

Values are median (IQR). Flow_mean_, mean flow; Flow_max_, maximum flow; V_mean_, mean velocity; V_max_, maximum velocity; WSS_mean_, mean wall shear stress on vascular endothelium; WSS_max_, maximum wall shear stress on vascular endothelium.

^*^Depending on angiographic characteristics of baseline digital subtraction angiography, the advanced stage was defined as Suzuki stages Ⅴ and Ⅵ.

^a^ Three-time point comparisons; ^b^ Baseline vs. postoperative week 1; ^c^ Baseline vs. 1-year follow-up; ^d^ Postoperative week 1 vs. 1-year follow-up.

**TABLE S7 The association between hemodynamic alterations of both carotid siphons and the clinical outcome at follow-up.**

|  | Good outcome  (N=16) | | | *P^a^* | *P^b^* | *P^c^* | *P^d^* | Excellent outcome  (N=19) | | | *P^a^* | *P^b^* | *P^c^* | *P^d^* |
| --- | --- | --- | --- | --- | --- | --- | --- | --- | --- | --- | --- | --- | --- | --- |
|  | Baseline | Postoperation | Follow-up |  |  |  |  | Baseline | Postoperation | Follow-up |  |  |  |  |
| Surgical side |  |  |  |  |  |  |  |  |  |  |  |  |  |  |
| Flow_mean_ (mL/s) | 2.12±1.67 | 2.15±1.81 | 1.67±1.36 | 0.010 | >0.999 | 0.012 | 0.046 | 2.11±1.40 | 2.02±1.46 | 1.71±1.21 | 0.032 | >0.999 | 0.044 | 0.052 |
| Flow_max_ (mL/s) | 2.92±2.31 | 2.98±2.41 | 2.33±1.88 | 0.015 | >0.999 | 0.023 | 0.064 | 3.00±1.93 | 2.94±2.12 | 2.45±1.64 | 0.019 | >0.999 | 0.027 | 0.037 |
| V_mean_ (cm/s) | 18.07±9.43 | 19.93±10.41 | 17.79±10.48 | 0.243 | - | - | - | 19.00±7.83 | 20.61±7.26 | 17.26±7.53 | 0.149 | - | - | - |
| V_max_ (cm/s) | 24.63±13.15 | 26.32±13.07 | 24.45±12.91 | 0.445 | - | - | - | 25.67±10.13 | 28.37±10.50 | 24.06±9.69 | 0.130 | - | - | - |
| WSS_mean_ (N/m^2^) | 0.025 (0.013-0.088) | 0.050 (0.020-0.078) | 0.035 (0.013-0.088) | 0.336 | - | - | - | 0.050 (0.010-0.100) | 0.060 (0.020-0.090) | 0.040 (0.020-0.100) | 0.339 | - | - | - |
| WSS_max_ (N/m^2^) | 0.090 (0.063-0.188) | 0.105 (0.063-0.175) | 0.100 (0.073-0.220) | 0.092 | - | - | - | 0.120 (0.080-0.210) | 0.140 (0.090-0.200) | 0.110 (0.080-0.180) | 0.559 | - | - | - |
| Contralateral side |  |  |  |  |  |  |  |  |  |  |  |  |  |  |
| Flow_mean_ (mL/s) | 2.98±1.60 | 2.89±1.57 | 2.50±1.46 | 0.008 | >0.999 | 0.008 | 0.173 | 2.69±2.11 | 2.87±2.04 | 2.35±1.80 | 0.039 | >0.999 | 0.256 | 0.016 |
| Flow_max_ (mL/s) | 4.03±2.14 | 3.90±2.17 | 3.37±1.95 | 0.010 | >0.999 | 0.008 | 0.188 | 3.62±2.66 | 3.95±2.80 | 3.27±2.47 | 0.012 | 0.590 | 0.256 | 0.011 |
| V_mean_ (cm/s) | 22.63±8.40 | 26.54±12.37 | 25.78±15.53 | 0.371 | - | - | - | 23.29±16.30 | 26.48±14.87 | 21.24±9.34 | 0.080 | - | - | - |
| V_max_ (cm/s) | 30.08±11.75 | 34.65±16.44 | 34.35±19.00 | 0.400 | - | - | - | 30.67±20.18 | 36.27±19.51 | 28.93±12.29 | 0.050 | - | - | - |
| WSS_mean_ (N/m^2^) | 0.050 (0.013-0.098) | 0.045 (0.020-0.093) | 0.065 (0.020-0.113) | 0.314 | - | - | - | 0.050 (0.020-0.090) | 0.060 (0.030-0.120) | 0.070 (0.020-0.100) | 0.385 | - | - | - |
| WSS_max_ (N/m^2^) | 0.125 (0.073-0.230) | 0.140 (0.093-0.215) | 0.130 (0.100-0.193) | 0.522 | - | - | - | 0.180 (0.150-0.250) | 0.180 (0.100-0.280) | 0.150 (0.080-0.190) | 0.418 | - | - | - |

Values are mean ± SD or median (IQR). Flow_mean_, mean flow; Flow_max_, maximum flow; V_mean_, mean velocity; V_max_, maximum velocity; WSS_mean_, mean wall shear stress on vascular endothelium; WSS_max_, maximum wall shear stress on vascular endothelium.

^a^ Three-time point comparisons; ^b^ Baseline vs. postoperative week 1; ^c^ Baseline vs. 1-year follow-up; ^d^ Postoperative week 1 vs. 1-year follow-up.

**TABLE S8 The association between hemodynamic alterations of both carotid siphons and the improvement of cerebral perfusion at follow-up.**

|  | Cerebral perfusion NOT improved  (N=12) | | | *P^a^* | *P^b^* | *P^c^* | *P^d^* | Cerebral perfusion improved  (N=23) | | | *P^a^* | *P^b^* | *P^c^* | *P^d^* |
| --- | --- | --- | --- | --- | --- | --- | --- | --- | --- | --- | --- | --- | --- | --- |
|  | Baseline | Postoperation | Follow-up |  |  |  |  | Baseline | Postoperation | Follow-up |  |  |  |  |
| Surgical side |  |  |  |  |  |  |  |  |  |  |  |  |  |  |
| Flow_mean_ (mL/s) | 2.48±1.82 | 2.66±1.87 | 2.25±1.48 | 0.105 | - | - | - | 1.92±1.31 | 1.78±1.41 | 1.40±1.05 | 0.001 | >0.999 | 0.002 | 0.017 |
| Flow_max_ (mL/s) | 3.45±2.51 | 3.69±2.56 | 3.21±2.03 | 0.208 | - | - | - | 2.71±1.82 | 2.57±1.98 | 1.97±1.42 | <0.001 | >0.999 | 0.001 | 0.008 |
| V_mean_ (cm/s) | 20.90±8.89 | 23.13±8.94 | 20.25±9.25 | 0.114 | - | - | - | 17.37±8.20 | 18.83±8.40 | 16.06±8.50 | 0.186 | - | - | - |
| V_max_ (cm/s) | 28.39±12.41 | 30.41±12.29 | 28.48±12.33 | 0.507 | - | - | - | 23.53±10.81 | 25.87±11.20 | 22.02±9.98 | 0.108 | - | - | - |
| WSS_mean_ (N/m^2^) | 0.050 (0.023-0.115) | 0.055 (0.033-0.080) | 0.080 (0.040-0.130) | 0.439 | - | - | - | 0.020 (0.010-0.090) | 0.050 (0.020-0.080) | 0.030 (0.010-0.060) | 0.507 | - | - | - |
| WSS_max_ (N/m^2^) | 0.110 (0.073-0.218) | 0.160 (0.095-0.220) | 0.190 (0.105-0.220) | 0.436 | - | - | - | 0.110 (0.070-0.190) | 0.110 (0.070-0.170) | 0.080 (0.070-0.150) | 0.545 | - | - | - |
| Contralateral side |  |  |  |  |  |  |  |  |  |  |  |  |  |  |
| Flow_mean_ (mL/s) | 2.59±1.53 | 2.75±1.62 | 2.34±1.36 | 0.048 | >0.999 | 0.360 | 0.010 | 2.95±2.05 | 2.94±1.94 | 2.46±1.78 | 0.010 | >0.999 | 0.018 | 0.038 |
| Flow_max_ (mL/s) | 3.62±2.12 | 3.78±2.27 | 3.22±1.89 | 0.040 | >0.999 | 0.241 | 0.008 | 3.91±2.59 | 4.01±2.66 | 3.37±2.41 | 0.008 | >0.999 | 0.016 | 0.038 |
| V_mean_ (cm/s) | 20.89±7.05 | 25.27±10.89 | 20.67±7.35 | 0.126 | - | - | - | 24.09±15.40 | 27.15±15.00 | 24.70±14.53 | 0.406 | - | - | - |
| V_max_ (cm/s) | 28.40±10.55 | 33.49±14.20 | 28.16±9.75 | 0.184 | - | - | - | 31.44±19.19 | 36.59±19.81 | 33.10±18.03 | 0.258 | - | - | - |
| WSS_mean_ (N/m^2^) | 0.050 (0.020-0.093) | 0.055 (0.023-0.093) | 0.065 (0.013-0.123) | 0.502 | - | - | - | 0.050 (0.020-0.090) | 0.050 (0.020-0.120) | 0.070 (0.020-0.100) | 0.319 | - | - | - |
| WSS_max_ (N/m^2^) | 0.180 (0.095-0.258) | 0.150 (0.108-0.243) | 0.140 (0.100-0.233) | 0.915 | - | - | - | 0.160 (0.090-0.210) | 0.150 (0.090-0.270) | 0.140 (0.100-0.190) | 0.632 | - | - | - |

Values are mean ± SD or median (IQR). Flow_mean_, mean flow; Flow_max_, maximum flow; V_mean_, mean velocity; V_max_, maximum velocity; WSS_mean_, mean wall shear stress on vascular endothelium; WSS_max_, maximum wall shear stress on vascular endothelium.

^a^ Three-time point comparisons; ^b^ Baseline vs. postoperative week 1; ^c^ Baseline vs. 1-year follow-up; ^d^ Postoperative week 1 vs. 1-year follow-up.

**TABLE S9 The association between hemodynamic alterations of both carotid siphons and postoperative collateralization at follow-up.**

|  | Grade A (N=12) | | | *P^a^* | *P^b^* | *P^c^* | *P^d^* | Grade B (N=13) | | | *P^a^* | *P^b^* | *P^c^* | *P^d^* |
| --- | --- | --- | --- | --- | --- | --- | --- | --- | --- | --- | --- | --- | --- | --- |
|  | Baseline | Postoperation | Follow-up |  |  |  |  | Baseline | Postoperation | Follow-up |  |  |  |  |
| Surgical side |  |  |  |  |  |  |  |  |  |  |  |  |  |  |
| Flow_mean_ (mL/s) | 1.76±1.30 | 1.88±1.62 | 1.28±1.06 | 0.009 | >0.999 | 0.041 | 0.040 | 2.19±1.55 | 1.95±1.52 | 1.69±1.27 | 0.091 | - | - | - |
| Flow_max_ (mL/s) | 2.38±1.67 | 2.59±2.13 | 1.76±1.33 | 0.009 | >0.999 | 0.055 | 0.050 | 3.04±2.15 | 2.78±2.18 | 2.38±1.74 | 0.079 | - | - | - |
| V_mean_ (cm/s) | 16.70±8.87 | 18.50±8.88 | 17.78±11.02 | 0.629 | - | - | - | 18.24±8.66 | 19.27±7.71 | 16.05±6.82 | 0.093 | - | - | - |
| V_max_ (cm/s) | 21.71±10.55 | 24.63±11.24 | 23.85±12.47 | 0.482 | - | - | - | 24.59±11.84 | 25.65±9.99 | 22.28±8.99 | 0.177 | - | - | - |
| WSS_mean_ (N/m^2^) | 0.020 (0.010-0.080) | 0.030 (0.013-0.080) | 0.025 (0.010-0.120) | 0.416 | - | - | - | 0.040 (0.010-0.075) | 0.060 (0.025-0.080) | 0.050 (0.030-0.085) | 0.458 | - | - | - |
| WSS_max_ (N/m^2^) | 0.100 (0.063-0.188) | 0.105 (0.065-0.160) | 0.090 (0.073-0.183) | 0.424 | - | - | - | 0.100 (0.060-0.175) | 0.140 (0.085-0.175) | 0.140 (0.085-0.195) | 0.186 | - | - | - |
| Contralateral side |  |  |  |  |  |  |  |  |  |  |  |  |  |  |
| Flow_mean_ (mL/s) | 2.61±2.12 | 2.63±1.89 | 1.97±1.60 | 0.052 | - | - | - | 3.02±1.77 | 3.08±1.74 | 2.58±1.55 | 0.016 | >0.999 | 0.039 | 0.065 |
| Flow_max_ (mL/s) | 3.25±2.38 | 3.53±2.55 | 2.61±2.08 | 0.024 | >0.999 | 0.070 | 0.050 | 4.16±2.39 | 4.18±2.41 | 3.51±2.12 | 0.010 | >0.999 | 0.031 | 0.051 |
| V_mean_ (cm/s) | 24.44±19.67 | 25.22±16.48 | 26.25±17.28 | 0.809 | - | - | - | 21.85±8.33 | 27.30±13.08 | 22.08±11.00 | 0.066 | - | - | - |
| V_max_ (cm/s) | 30.77±23.82 | 33.39±21.88 | 34.42±21.17 | 0.688 | - | - | - | 29.10±11.50 | 36.07±17.00 | 29.48±14.28 | 0.063 | - | - | - |
| WSS_mean_ (N/m^2^) | 0.050 (0.023-0.078) | 0.045 (0.023-0.155) | 0.080 (0.013-0.145) | 0.826 | - | - | - | 0.050 (0.015-0.105) | 0.050 (0.020-0.070) | 0.050 (0.020-0.105) | 0.199 | - | - | - |
| WSS_max_ (N/m^2^) | 0.140 (0.083-0.210) | 0.120 (0.083-0.288) | 0.145 (0.090-0.243) | 0.853 | - | - | - | 0.160 (0.100-0.180) | 0.130 (0.095-0.210) | 0.150 (0.085-0.190) | 0.763 | - | - | - |

**TABLE S9 (continue).**

|  | Grade C (N=10) | | | *P^a^* | *P^b^* | *P^c^* | *P^d^* |
| --- | --- | --- | --- | --- | --- | --- | --- |
|  | Baseline | Postoperation | Follow-up |  |  |  |  |
| Surgical side |  |  |  |  |  |  |  |
| Flow_mean_ (mL/s) | 2.44±1.73 | 2.49±1.80 | 2.18±1.40 | 0.124 | - | - | - |
| Flow_max_ (mL/s) | 3.57±2.42 | 3.63±2.46 | 3.18±1.97 | 0.166 | - | - | - |
| V_mean_ (cm/s) | 21.26±7.92 | 23.80±9.55 | 19.05±8.96 | 0.060 | - | - | - |
| V_max_ (cm/s) | 30.16±11.29 | 33.11±13.07 | 27.25±12.34 | 0.093 | - | - | - |
| WSS_mean_ (N/m^2^) | 0.090 (0.020-0.138) | 0.060 (0.025-0.090) | 0.035 (0.018-0.123) | 0.900 | - | - | - |
| WSS_max_ (N/m^2^) | 0.170 (0.093-0.253) | 0.140 (0.085-0.225) | 0.120 (0.068-0.210) | 0.023 | 0.221 | 0.030 | >0.999 |
| Contralateral side |  |  |  |  |  |  |  |
| Flow_mean_ (mL/s) | 2.82±1.86 | 2.90±1.97 | 2.76±1.80 | 0.645 | - | - | - |
| Flow_max_ (mL/s) | 4.01±2.60 | 4.07±2.74 | 3.91±2.48 | 0.769 | - | - | - |
| V_mean_ (cm/s) | 22.73±8.90 | 27.01±11.57 | 21.40±7.22 | 0.026 | 0.109 | >0.999 | 0.148 |
| V_max_ (cm/s) | 31.64±13.06 | 37.40±15.28 | 30.30±9.67 | 0.027 | 0.112 | >0.999 | 0.124 |
| WSS_mean_ (N/m^2^) | 0.060 (0.018-0.108) | 0.075 (0.028-0.105) | 0.075 (0.028-0.100) | 0.814 | - | - | - |
| WSS_max_ (N/m^2^) | 0.235 (0.133-0.253) | 0.170 (0.140-0.263) | 0.130 (0.108-0.198) | 0.091 | - | - | - |

Values are mean ± SD or median (IQR). Flow_mean_, mean flow; Flow_max_, maximum flow; V_mean_, mean velocity; V_max_, maximum velocity; WSS_mean_, mean wall shear stress on vascular endothelium; WSS_max_, maximum wall shear stress on vascular endothelium.

^a^ Three-time point comparisons; ^b^ Baseline vs. postoperative week 1; ^c^ Baseline vs. 1-year follow-up; ^d^ Postoperative week 1 vs. 1-year follow-up.
